# Executive Function in Relation to White Matter in Preterm and Full Term Children

**DOI:** 10.3389/fped.2018.00418

**Published:** 2019-01-15

**Authors:** Irene M. Loe, Jenna N. Adams, Heidi M. Feldman

**Affiliations:** ^1^Department of Pediatrics, Stanford University, Stanford, CA, United States; ^2^Helen Wills Neuroscience Institute, University of California, Berkeley, Berkeley, CA, United States

**Keywords:** preterm birth, premature birth, executive function, diffusion tensor imaging, white matter, working memory

## Abstract

**Background:** Executive function (EF) refers to cognitive abilities used to guide goal-directed behavior. Diffusion Tensor Imaging (DTI) provides quantitative characterization of white matter tracts in the brain. Children with preterm birth often have EF impairments and white matter injury.

**Aim:** To examine the degree of association between EF scores and white matter fractional anisotropy (FA) as measured by DTI in children born preterm and term

**Study design:** Cross-sectional study

**Subjects:** Participants, 9–16 years of age, born preterm (*n* = 25; mean gestational age 28.6 weeks; mean birth weight 1,191 grams), and full term (*n* = 20)

**Outcome measures:** White matter FA analyzed with Tract-Based Spatial Statistics, a technique that generates a skeleton representing the core of white matter tracts throughout the brain. Behavioral scores from EF tasks examining working memory, spatial memory capacity, and multiple skills from the Stockings of Cambridge.

**Results:** The groups performed comparably on all tasks. In both groups, unfavorable working memory strategy scores were associated with lower FA. Other measures of EF were not associated with whole skeleton FA in either group in either direction.

**Conclusions:** Strategy score on a spatial working memory task was associated with FA in preterm and full term children, suggesting common underlying neurobiology in both groups. Associations were found in frontal-parietal connections and other major tracts. Lack of associations between other EF tasks and FA may be due to variation in how children accomplish these EF tasks. Future research is required to fully understand the neurobiology of EF in children born preterm.

## Introduction

Executive Function (EF) refers to interrelated cognitive abilities (i.e., working memory, response inhibition, cognitive flexibility, and organization/planning) used to guide goal-directed behavior (1). EF skills are associated with academic achievement and social competence (2–4). Functional neuroimaging studies link EF skills to prefrontal, parietal, and temporal cortices (5–7). EF performance has also been associated with white matter connections among brain regions (8, 9).

EF impairments are common in preterm children (10, 11). They are attributed, in part, to white matter injury (8, 9), which is common after preterm birth (12–15). Associations have been reported between EF skills in childhood and newborn white matter abnormalities on conventional magnetic resonance imaging (MRI) (16–19), which provides a qualitative impression of white matter properties.

Diffusion tensor imaging (DTI) generates quantitative measures to characterize white matter (20). Fractional anisotropy (FA) measures the degree to which diffusion is anisotropic, reflecting directional coherence of axons within a white matter tract (21, 22). Reduced FA in preterm children compared to full term children has been found in multiple brain regions in the absence of cystic lesions (12, 14, 15) and has been used to infer subtle injury, though not all studies find differences in all tracts (23–25). Very few DTI studies of preterm children have focused on EF skills and white matter properties. One DTI study of very low birth weight adolescents found that low FA in the left cingulum and bilateral inferior fronto-occipital fasciculi (IFOF) was significantly correlated with low EF (9). Another DTI study found that diffusivity measures other than FA at term-equivalent age in the inferior occipital and cerebellar regions were associated with impaired EF at age 7 years (26). In contrast, a DTI study of 8-year-old very preterm children found no association between DTI measures and parent-rated EF (27).

Techniques such as resting state functional MRI and structural connectivity may also be combined to better understand the relationship between white matter maturation and the development of functional connectivity among brain networks (28, 29). A study of typical development between ages 2 and 18 years found positive correlations between structural and functional connectivity and that the relationship strengthened with age (28). A study utilizing diffusion MRI with whole-brain mapping of connectivity in 11- to 31-month-old preterm infants found altered connection strength associated with age and preterm birth (30). Older children had stronger connections in tracts involving frontal lobe structures, whereas increased prematurity at birth was related to widespread reductions in connection strength in tracts involving all cortical lobes and several subcortical structures (30).

This exploratory study used DTI in preterm children and adolescents to examine associations between EF skills and FA using Tract-Based Spatial Statistics (TBSS), a whole-brain, mean white matter FA skeleton approach. We focused on FA rather than other DTI metrics, such as axial diffusivity (AD) or radial diffusivity (RD), since both AD and RD are reflected in FA (i.e., FA = AD/RD^2^). In this sample, we previously reported group differences in FA (25), and significant associations were found between white matter FA in multiple regions and macroscopic injury (25), IQ (25), language (31), and parent-reported behavior symptoms (32). Therefore, we hypothesized that we would find significant associations between measures of EF skills and white matter FA. We suspected significant correlations in tracts that connect frontal and parietal regions, such as the superior longitudinal fasciculus and the arcuate fasciculus, given that other studies have found associations in these tracts or activations in frontal and parietal regions (33–36).

## Methods

### Participants

Participants were 9–16 year olds, born between 1992 and 1999, and participating in a larger multi-site study (37). The study was carried out in accordance with the recommendations of the Belmont Report, released by the National Commission for the Protection of Human Subjects in Biomedical and Behavioral Research. The protocol (#6985) was approved by the Stanford University IRB. All parents or guardians gave written informed consent and child and adolescent participants gave assent in accordance with the Declaration of Helsinki. Preterm subjects (*n* = 25) were born at ≤34 weeks gestation with birth weight <2,500 grams. Controls were born ≥37 weeks (*n* = 20). Participants included in the current study are those who had complete EF data and concurrent DTI data. Due to protocol changes in the larger study, not all subjects completed EF testing, resulting in a smaller number of controls. Exclusions included seizures; hydrocephalus; receptive vocabulary score <70; hearing loss; and non-English speaker. Controls were also excluded for language, learning, or psychiatric disorders. Three preterm subjects were excluded due to extremely enlarged ventricles (38). Preterm subjects were recruited by letters to families previously evaluated at the neonatal follow-up clinic. Controls were recruited through ads and group-matched to preterm children for age, sex, and race. There were no differences in maternal education, IQ scores, or handedness. See Table 1. Medical complications are in Supplemental Material.

**Table 1:** Participant demographics and behavior scores (*N* = 45).

**Participant Characteristics**	**Preterm (*****n*** **=** **25)**		**Full Term (*****n*** **=** **20)**			
	**Number (*n*)**	**%**	**Number (*n*)**	**%**	***X*^**2**^**	***p***
Boys	12	48	10	50	0.894	0.566
Maternal Education <High School	3	12	5	25	0.435	0.229
Non-white	8	32	10	50	1.5	0.221
Left-handed or ambidextrous	4	16	4	20	0.081	0.775
Special education	7	28	0	0	6.63	0.010^*^
	**Mean (SD)**	**Range**	**Mean (SD)**	**Range**	***t***	***p***
Age (years)	12.7 (2.1)	9.7–16.8	12.9 (2.1)	9.4–16.9	0.203	0.654
IQ	109 (15.8)	72–136	113 (16.1)	86–142	0.759	0.388
Gestational Age (weeks)	28.6 (2.5)	26–34	39.3 (1.1)	37–40	300	<0.001^*^
Birth Weight (grams)	1191 (466)	630–2410	3171 (403)	2438–3771	226	<0.001^*^
**EXECUTIVE FUNCTION OUTCOMES**						
**Spatial Working Memory**						
Strategy Score	31.6 (5.7)	20–40	33.8 (5.1)	20–40	1.37	0.178
Forgetting Errors	24.3 (17.1)	0–50	23.9 (13.9)	4–49	−0.08	0.936
**Spatial Span**						
Span Length	6.4 (1.3)	4–9	6.7 (1.6)	3–9	0.62	0.539
**Stockings of Cambridge**						
Problems Solved	7.9 (2.4)	3–12	9.0 (1.9)	6–12	1.71	0.094

* *indicates significance at p <0.05*.

### Executive Function Measures

We utilized the Cambridge Neuropsychological Test Automated Battery (Cambridge Cognition, Ltd, Cambridge, UK), a computerized EF battery used in studies of preterm children (39). Spatial Working Memory (SWM), Spatial Span (SS), and Stockings of Cambridge (SOC) subtests captured different EF constructs. SWM measures the ability to retain and manipulate spatial information. Two outcomes include *Strategy score* [a low value indicates a systematic approach (40)] and *Forgetting errors*. SS measures spatial capacity and is a visual-spatial analog of the digit span task. *Span length* is the longest sequence successfully recalled. SOC measures response inhibition, planning, and organization skills. A high value on *Problems solved* indicates good planning/organization skills. Additional details are in Supplemental Material.

### Statistical Analysis

Chi-square, independent *t*-tests, and Mann-Whitney *U* tests were used to evaluate between-group differences on demographic variables and outcome measures.

### Imaging Protocols

Details for MRI and DTI protocols/processing were previously reported (32) and are in Supplemental Material. MRI data were acquired on a 3T Signa Excite (GE Medical Systems, Milwaukee, WI). For DTI, a diffusion-weighted, single-shot, spin-echo, echo-planar imaging sequence was used to acquire 60 slices, 2 mm thick, in 30 different diffusion directions (b = 900).

### DTI Image Analysis and Post-processing

We used the voxel-wise “Tract Based Spatial Statistics” (TBSS) method from the Oxford Center for Functional MRI of the Brain (FMRIB) Diffusion Toolbox (41). TBSS identifies a core white matter “skeleton” that is anatomically equivalent across subjects. Advantages of TBSS include avoiding spatial smoothing (averaging voxels) and minimizing partial volume effects that can occur when more than one tract goes through a voxel, thereby leading to decreases in anatomic specificity (41). We analyzed FA, a ratio from 0 to 1.

A tensor model was fit to each voxel (42), and FA images were calculated. FA images were registered to the most representative subject in the study (target) using the FMRIB Software Library's (FSL) non-linear registration tool (43). Each subject was aligned to the target; aligned FA images were averaged to create a mean FA map. An FA threshold ≥0.2 was designated to include major white matter pathways and to exclude peripheral tracts. The mean FA image was generated and thinned to create the mean FA-skeleton, a representation of the core of all major white matter tracts common to the group. For each subject, TBSS projected the highest FA on to the FA-skeleton, corresponding to the local tract core.

### Statistical Analysis of DTI Data

Analysis of the FA values at the voxel level was conducted using a permutation-based tool for non-parametric statistical thresholding (“Randomize” function in FSL) to assess group related differences (44). This method generates non-parametric, two-sample, unpaired *t*-tests of FA in subjects compared with controls. We assessed the degree of association between EF scores and FA of the mean skeleton within the preterm group and the control group separately in both the positive and negative directions as well as group by task interactions, covarying by age. We set significance at *p* < 0.05 after correction for multiple comparisons using Threshold-Free Cluster Enhancement, a method that avoids using an arbitrary threshold for initial cluster-formation or identification of signal (45). Significant clusters of voxels on the FA-skeleton were inspected with reference to John Hopkins University DTI-based white matter atlases (46) to determine tract assignments.

## Results

No significant behavioral differences were found between groups on raw scores on any EF measure (Table 1). On voxel-wise analysis, for both preterm and full term groups, SWM strategy scores were significantly negatively associated with the mean FA-skeleton; as strategy scores increased (indicating poor performance), FA decreased. There were no significant positive correlations. There were no significant associations in either direction for the other measures in either group. We also examined interaction effects across both groups; there were no significant group by task interactions.

**Figure 1:**
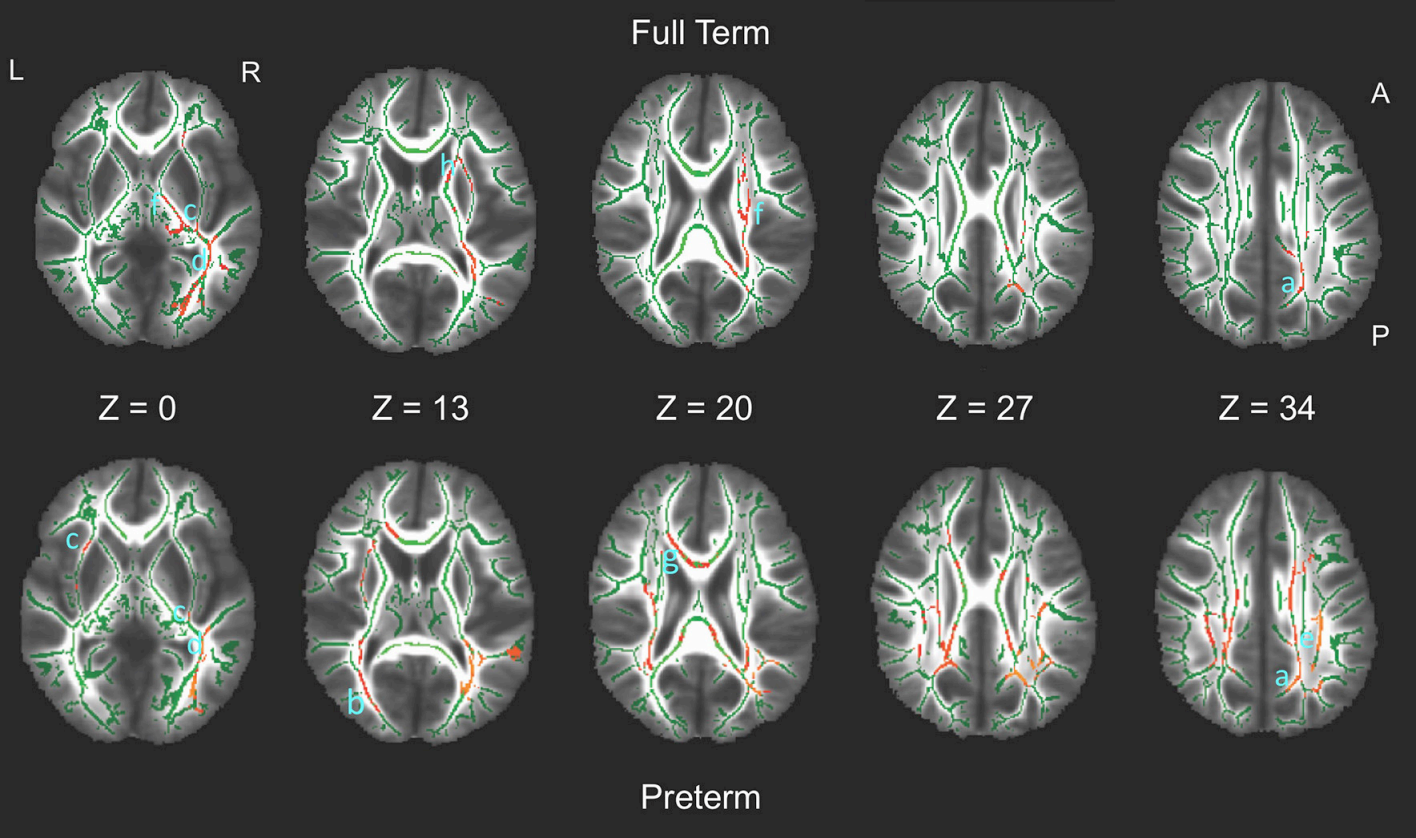
White matter regions on the fractional anisotropy (FA) skeleton (green) of statistically significant associations between FA and spatial working memory strategy score in full term (upper row, red) and preterm (bottom row, red) groups (*p* < 0.05, non-parametric permutation test, corrected for multiple comparisons) overlaid on axial slices of the mean FA image (grayscale). Tracts in which significant associations were found include: Both groups: a. cingulum; b. forceps major; c. inferior fronto-occipital fasciculus; d. inferior longitudinal fasciculus; e. superior longitudinal fasciculus; f. corticospinal tract. Preterm group only: g. corpus callosum/forceps minor. Full term group only: h. anterior thalamic radiation. L, left; R, right; A, anterior; P, posterior.

In both groups, tracts associated with strategy score included the superior longitudinal fasciculus (SLF), cingulum, inferior fronto-occipital fasciculus (IFOF), inferior longitudinal fasciculus (ILF), forceps major, and corticospinal tracts. The preterm group also had associations with the corpus callosum and forceps minor, whereas full term children had additional associations with the anterior thalamic radiations. Figure 1 shows the multiple regions of significant association on axial views of the FA-skeleton for the full term group (upper row) and the preterm group (lower row).

## Discussion

In summary, we found that FA in multiple tracts was associated with a single measure of EF—strategy score. Lower FA was associated with poorer strategy in both preterm and full term groups. Similar tracts were identified for both groups, although unique tracts were also associated with each group.

Our results are similar to another report of associations between lower FA and EF measures of problem solving and cognitive flexibility in a preterm sample within the cingulum and bilateral IFOF (9). The cingulum bundles course from the medial temporal lobe to the frontal lobes, and frontal regions have long been implicated in EF studies of typical adults (47, 48) and clinical populations using DTI (49–51). The IFOF courses from the occipital lobes through the external capsules to the inferior frontal cortex. A study of very preterm children at age 7 years found that diffusivity measures other than FA at term-equivalent age in similar inferior occipital regions were associated with poor planning ability (26). The associations with these tracts may reflect the importance of visual input to frontal regions during processing of the task.

The association between FA and strategy score in both preterm and full term groups suggests a common neurobiological basis for memory strategy. In both groups, FA in the SLF was also associated with strategy score. These findings are similar to those in a study of typical children age 7–13 years that used the same CANTAB Spatial Working Memory task (34). Another study of typical children, ages 5–17 years, found that higher FA in the SLF was correlated with better set shifting/cognitive flexibility and inhibition (52).

Associations of FA and strategy score were also found within the ILF, forceps major, and corticospinal tracts for both groups. The ILF courses from the occipital to temporal lobes. Adult DTI studies showed associations between the ILF and a memory composite score in healthy adults (48) and working memory in adults with diffuse axonal injury (49). The forceps major extends from the corpus callosum to connect homologous regions of the occipital lobe in each hemisphere. A tractography study of healthy aging in adults found associations between the splenium of the corpus callosum and memory and EF (53). The corticospinal tracts are associated with motor domains; associations may reflect motor planning and actions required to perform the task.

In the preterm group, the corpus callosum and forceps minor were also significantly associated with SWM strategy score. The corpus callosum provides bi-hemispheric connections, and the forceps minor extend from the corpus callosum to connect homologous regions of the anterior frontal lobe in each hemisphere. A study of healthy 8–18 year olds found correlations between FA in frontal-parietal connections and neural activation on functional MRI in the superior frontal sulcus and inferior parietal lobe (54). In the full term group, the anterior thalamic radiations were identified. The ATR connects subcortical regions to the frontal lobe. A DTI study of patients with first-episode psychosis found associations of decreased FA in the ATR, among other regions, and impairments on a measure of cognitive flexibility (55). Subcortical-frontal connections are also important for EF in other DTI studies of children with Attention Deficit Hyperactivity Disorder (ADHD) (56, 57).

We did not find associations between FA and SS or SOC in either the preterm or full term groups. These findings are in sharp contrast with other studies using the same group of participants and methods (25, 31, 32). These studies identified many positive associations between FA and other domains of function in PT children, including intelligence, language (i.e., linguistic processing speed), reading (i.e., syntactic comprehension and decoding), and behavior problems (i.e., inattention and internalizing symptoms) (25, 31, 32). The tracts associated with SWM strategy show some overlap with those associated with IQ, language, reading, and behavior symptoms, although the areas of correlation with EF are less widespread than those seen with language, reading and behavior outcomes. The reasons for the lack of findings with EF are unclear. Children may use a variety of different strategies for these tasks that differ across groups and/or represent higher individual variation within groups. These strategies may also vary with age and may have further increased the heterogeneity in this sample with wide age range. If these possibilities are true, the associations of EF skills and white matter of any specific tracts may be limited. Children may approach working memory strategy more consistently than they approach other EF skills. The lack of findings with other EF skills may also reflect methodological details. SWM strategy has a greater range than SS total span length or problems solved on the SOC. It is easier to detect correlations when the range of scores is high. Another possibility relates to limitations of TBSS, which focuses on the centers of white matter tracts and may not detect peripheral damage.

## Limitations

The study utilized a small, heterogeneous convenience sample of preterm children who may not be representative of all preterm children. The EF tasks did not differentiate the groups behaviorally. The wide age range may have limited the ability to find brain-behavior relationships and group differences, as the groups may have different developmental trajectories for EF skills in middle childhood and early adolescence.

## Conclusion

Use of a systematic strategy on a spatial working memory task was correlated with white matter FA in preterm and full term children, suggesting common underlying neurobiology in both groups. Better performance was associated with higher FA. Associations were found in frontal-parietal connections and other major tracts. Our study adds to the relatively small body of literature on EF and white matter in preterm children. Replication with a larger, more homogeneous sample may provide insight into the neurobiology underlying EF in children in general. Unexpectedly, no other associations between EF scores and FA were found in either group. Future work should focus on smaller age groups and adjust tasks and DTI methodology, including advanced imaging and analysis methods such as combined functional MRI and structural connectivity, to better understand the neurobiology of EF.

## Author Contributions

IL conceptualized the study, analyzed and interpreted data, drafted the initial manuscript and critically reviewed, and revised the manuscript. JA contributed to conceptualization and interpretation of data, literature review, and critically reviewed and revised the manuscript. HF conceptualized the study, analyzed and interpreted data, and critically reviewed and revised the manuscript. All authors are responsible for the reported work and have approved the final manuscript.

### Conflict of Interest Statement

The authors declare that the research was conducted in the absence of any commercial or financial relationships that could be construed as a potential conflict of interest.

## Abbreviations

AD: axial diffusivity
ADHD: attention deficit hyperactivity disorder
DTI: diffusion tensor imaging
EF: executive function
FA: fractional anisotropy
FMRIB: functional magnetic resonance imaging of the brain
FSL: FMRIB Software Library
IFOF: inferior fronto-occipital fasciculus
ILF: inferior longitudinal fasciculus
MRI: magnetic resonance imaging
RD: radial diffusivity
SLF: superior longitudinal fasciculus
SOC: Stockings of Cambridge
SS: Spatial Span
SWM: spatial working memory
TBSS: tract based spatial statistics.
