# Supplementary material for: Executive Function in Relation to White Matter in Preterm and Full Term Children
Source: Front Pediatr. 2019 Jan 15;6:418. doi: 10.3389/fped.2018.00418 (PMC6341022; doi:10.3389/fped.2018.00418)
Supplement: Supplementary file 1 [file Data_Sheet_1.PDF]

Supplemental Material

Participant Characteristics:

Medical complications at birth in the preterm group: 4 of the 9 subjects on whom we had perinatal imaging results had abnormal findings on head ultrasounds or MRIs ($\geq$ grade 2 IVH, echodensities, or cystic lesions); 2 had mildly abnormal findings (grade 1 hemorrhage or choroid plexus cyst); 13 had RDS; 5 developed bronchopulmonary dysplasia; and 2 were small for gestational age ($\leq 3^{rd}$ percentile). We had current imaging results on all children at the time of the study. We could not analyze behavioral outcomes in relation to perinatal imaging scans because not all children were scanned in the perinatal period at our institution.

Methods:

Executive Function Measures

CANTAB tasks:

1. SWM measures the ability to retain and manipulate spatial information. Colored boxes are shown on the screen. Participants find hidden tokens in trials with four, six, and eight boxes. The computer hides one token at a time and uses each box only once in a set. Participants use a process of elimination to find all the tokens. A low value on *strategy score* indicates a systematic approach, usually a predetermined search sequence to reduce memory load. *Total errors score* is comprised of the number of times the child touched boxes that have been found to be empty or revisited boxes that have already been found to contain a token.
2. SS measures spatial capacity and is a visual-spatial analogue of the digit span task. Participants are presented an array of 2 to 9 squares that briefly change color one at a time; subjects then touch the squares in the same order. *Span length* is the longest sequence successfully recalled.
3. SOC measures response inhibition, planning, and organization skills. Participants rearrange colored balls into specific patterns, moving one ball at a time, to copy presented patterns in the least number of moves. A high value on Problems Solved in Minimum Moves (*problems solved*) indicates good planning and organization skills.

MRI acquisition protocol

MRI data were acquired on a 3T Signa Excite (GE Medical Systems, Milwaukee, WI). Three T1 images were acquired and averaged for anatomical registration. Two high resolution IR-prep 3D FSPGR scans (FOV = 24 x 18 cm, matrix size = 260 x 192, 0.9 mm slices, TI = 300 ms, flip angle = 15 degrees, 1 NEX) and one IR-prep 3D FSPGR scan (FOV = 24 x15.6 cm, matrix size = 256 x 192, 1.2mm slices, TI = 300 ms, flip angle = 15 degrees, 1 NEX) were collected. The three T1 images were averaged for anatomical registration. A trained experimenter manually identified the anterior and posterior commissures and mid-sagittal plane, and these points were used to put the image in a canonical orientation.

DTI protocol, image analysis and post-processing

For DTI, a diffusion-weighted, single-shot, spin-echo, echo-planar imaging sequence (TE = 80 ms, TR = 6500 ms, FOV = 240 mm, matrix size = 128 x 128) was used to

acquire 60 slices, 2 mm thick, in 30 different diffusion directions (b = 900). The sequence was repeated 4 times, and 10 non-diffusion weighted (b = 0) volumes were collected.

We used the voxel-wise "Tract Based Spatial Statistics" method from the Oxford Center for Functional MRI of the Brain (FMRIB) Diffusion Toolbox. This method focuses on differences in white matter voxels by identifying a core white matter "skeleton" that is anatomically equivalent across subjects. Tract Based Spatial Statistics avoids spatial smoothing (averaging voxels) and minimizes partial volume effects that can occur when more than one tract goes through a voxel, thereby leading to decreases in anatomical specificity. We analyzed fractional anisotropy (FA), a ratio from 0 to 1 that indexes the magnitude of diffusion in one direction compared to other directions. FA is elevated in white matter in comparison to gray matter and cerebral spinal fluid.

Standard preprocessing began with correction of the DTI images for eddy current distortions in the data acquisition by affine registration to a non-diffusion weighted volume. A tensor model was fit to each voxel and FA images were calculated. Due to variation in white matter tracts across individuals, the FA images were registered to a FA target image in standard space. The FA image of the most representative subject in the study (target) was chosen based on the minimum necessary warping, using the FMRIB Software Library's (FSL) nonlinear registration tool. The target image was sampled to 1 mm^3 resolution and aligned by affine transformation to the Montreal Neurological Institute template (MNI152), allowing us to use standard space coordinates for describing regions. Then, each subject's image was aligned to the target. We found no differences between preterm and control groups in the degree of non-linear warping using independent samples *t*-test.

The aligned FA images were averaged to create a mean FA map. We set a threshold of FA $\geq$ .2 to include major white matter pathways and to exclude peripheral tracts where there would be significant inter-subject variability and/or considerable mixing of grey and white matter. The mean FA image was generated and thinned to create the mean FA-skeleton, a representation of the centers or core of all major white matter tracts common to the group. Finally, for each subject, TBSS projected the highest FA on to the FA-skeleton, corresponding to the local tract core or center value. The resulting data were available for voxel-wise cross-subject statistics.
